# The Role of Ets Factors in Tumor Angiogenesis

**DOI:** 10.1155/2010/767384

**Published:** 2010-05-04

**Authors:** Peter Oettgen

**Affiliations:** Division of Cardiology, and Molecular and Vascular Biology, Department of Medicine, Research North RM 270D, Beth Israel Deaconess Medical Center, 330 Brookline Avenue, Boston, MA 02115, USA

## Abstract

Angiogenesis is a critical component of tumor growth. A number of growth factors, including VEGF, FGF, and HGF, have been implicated as angiogenic growth factors that promote tumor angiogenesis in different types of cancer. Ets-1 is the prototypic member of the Ets transcription factor family. Ets-1 is known to be a downstream mediator of angiogenic growth factors. Expression of Ets-1 in a variety of different tumors is associated with increased angiogenesis. A role for other selected members of the Ets transcription factor family has also been shown to be important for the development of tumor angiogenesis. Because Ets factors also express a number of other important genes involved in cell growth, they contribute not only to tumor growth, but to disease progression. Targeting Ets factors in mouse tumor models through the use of dominant-negative Ets proteins or membrane permeable peptides directed at competitively inhibiting the DNA binding domain has now demonstrated the therapeutic potential of inhibiting selected Ets transcription factors to limit tumor growth and disease progression.

Ets-1 is the prototypic member of the Ets transcription factor family [1]. Several studies have demonstrated a role for Ets transcription factors in the regulation of endothelial-specific genes including VEGF-R1, VEGF-R2, Tie1, and Tie2. Whereas, the Ets factors Ets-1 and Ets-2 potently transactivate the Flt-1 gene promoter, they do not appear to regulate the Tie1 or Tie2 gene promoters [2, 3]. Ets-1 has been shown to cooperate with HIF-2*α* in the setting of hypoxia to regulate the expression of the VEGF-R2 [4, 5]. In contrast, the Ets factor ELF-1 is a potent transactivator of the Tie1 and Tie2 genes [2, 3]. ELF-1 has been shown to regulate other genes involved in angiogenesis including angiopoietin-2 and endothelial nitric oxide synthase [6, 7]. Ets-2 regulates the expression of CD13/aminopeptidase N (APN) in human endothelial cells [8]. Knockdown of Ets-2 in human endothelial cells using siRNA oligonucleotides significantly reduced the ability of the endothelial cells to form tubes and capillary networks. The selectivity of the different Ets factors to transactivate different target genes also correlates with their ability to bind to specific conserved Ets binding sites within these genes. However, when the DNA binding domain is highly homologous, as is the case for Ets-1 and Ets-2, there may be a significant overlap with respect to their downstream target genes. 

Ets factors can be subcategorized into subfamilies based on DNA and protein sequence homology. For example, Elf-1, Nerf-2, and Mef are highly related and belong to one subfamily. Similarly Fli1 and ERG constitute another subfamily, as do Ets1 and Ets2. The subfamily members often have overlapping functions [9]. From an evolutionary standpoint, this built-in redundancy may be protective against the effects of genetic mutations of the individual family members for critical developmental processes such as vasculogenesis. The DNA binding domain of the Ets family of proteins is a highly conserved region of approximately 85 residues that shares a strong sequence homology and three-dimensional structural scaffold that closely mimics the helix-turn-helix family of DNA binding proteins [10, 11]. Previous investigations have clearly established that the several highly conserved residues that are localized within the recognition helix, H3, are responsible for anchoring the Ets domain through requisite DNA contacts within the major groove. However, residues within the turns separating helices H2 and H3 and the first two *β*-strands are also involved in critical phosphate backbone contacts within the DNA minor groove. 

Expressions of selected Ets factors are also enriched at sites of active blood vessel development during embryogenesis. The Ets transcription factor Fli-1 is enriched in the developing blood vessels of zebrafish embryos [12]. ELF-1 is highly expressed in extra-embryonic and embryonic blood vessels of the developing mouse and chicken embryos [7, 13]. The Ets factor Ets-1 is also enriched in the developing blood vessels of the chicken, and antisense oligonucleotides have been shown to inhibit angiogenesis when delivered to the chicken chorioallantoic membrane [14]. Homozygous inactivation of Ets1 is associated with abnormalities in T cell function, but with no defects in vascular development or angiogenesis [15]. Similarly, while Ets2 is a critical regulator of trophoblast function during extraembryonic development it is dispensable for development of the embryo proper [16]. Mice with a homozygous hypomorphic mutant of Ets2, in which the conserved threonine 72 phosphorylation site is mutated, and Ets2^T72A/T72A^ mice are viable and appear normal [17]. When both Ets-1 and Ets-2 were simultaneously targeted, this led to striking defects in vascular development [18].

In addition to their role in regulating endothelial cell restricted genes, Ets factors have also been shown to function upstream and downstream of a number of angiogenic growth factors [19]. Overexpression of Ets-1 in tissues is associated with increased expression of both vascular endothelial growth factor (VEGF) and hepatocyte growth factor (HGF). Neutralizing antibodies to VEGF and HGF markedly attenuate the angiogenic effects of overexpressing Ets-1. Ets-1 regulates the expression of several downstream targets in endothelial cells that promote an angiogenic phenotype including the VEGF receptors (VEGF-R1 and VEGF-R2), urokinase, and several matrix metalloproteinase's (MMPs) including MMP-1, MMP-3, MMP-9 (Figure 1). Interestingly, HGF is also capable of inducing the expression of Ets-1, thereby creating a positive feedback loop at sites where either Ets-1 or HGF is expressed. In addition to HGF, fibroblast growth factor (FGF) and VEGF gene expression are regulated by Ets-1 [20, 21]. More recently, the chemokine CCL2 (MCP-1/JE) has been shown to promote angiogenesis and one of the main downstream effectors is Ets-1 [22]. The particular downstream targets identified in response to CCL2 in endothelial cells are the *β*3 integrins. Ets-1 is also involved in the regulation of CCL2 expression [23].

Several studies support a role for Ets-1 expression in the development of tumor angiogenesis in many different types of human cancer. Increased expression of Ets-1 in tumors is often associated with a worse prognosis (summarized in Table 1). In ovarian cancer, for example, Ets-1 expression strongly correlates with the degree of angiogenesis in the primary tumor and the development of metastatic lesions [24]. The prognosis is also significantly worse in those patients with high levels of Ets-1 expression in the tissues derived from the primary cancer. The survival rate of patients with histologically demonstrated high levels of Ets-1 expression at 24 months was 30% whereas that of patients with low levels was 70% [25]. These statistics support the overall concept that Ets-1 expression in ovarian cancers contributes to tumor growth and progression that is at least in part mediated by an increase in the degree of angiogenesis. Additional factors promoting tumor growth and progression are discussed below.

Esophageal squamous cell carcinoma (ESCC) is associated with a late presentation due to limited symptoms early in the disease. However, once detected, ESCC is a highly aggressive tumor with a propensity to metastasize. Ets-1 is overexpressed in 44/55 (80%) of tumor tissues obtained from patients with ESCC [26]. VEGF expression was observed in most of the Ets-1 overexpressing tissues and was strongly correlated with lymphnode metastasis. Kaplan-Meier survival analysis for patients with concomitant tissue expression of Ets-1 and VEGF demonstrated a significantly worse disease free survival. This again supports the concept that increased angiogenesis in Ets-1 expressing tumors is associated with increased tumor progression and a worse prognosis. 

VEGF and Ets-1 are also highly expressed in breast carcinoma [27]. Ets-1 is expressed in 53% of tumors of patients with newly diagnosed breast cancer. Ets-1 overexpression in breast cancer is associated with more invasive tumors and a significantly poorer prognosis [28]. At 5 years, less than 20% of patients with Ets-1 negative tumors have relapsed, compared to nearly 50% of those with Ets-1 expressing tumors. The expression of Ets-1 strongly correlated with the microvessel density within the tumors and lymph node involvement. In a study of 123 patients with primary breast cancer followed for 62 months, Ets-1 expression correlated strongly with VEGF expression and was an independent predictor of poor prognosis [28]. As a result of these findings, antiangiogenic therapy is currently being evaluated as adjunctive therapy for breast cancer. 

In a study of 60 patients with uterine cancer a strong Ets-1 expression was observed in 66% of patients, and was associated with increased tumor microvessel density [34]. In patients with cervical carcinoma, high Ets-1 expression was observed in tissues obtained from 25/60 (42%) patients and was also associated with a high microvessels density [30]. Furthermore, the 24-month survival rate of patients with high ets-1 expression was significantly worse at 54% compared to 92% for those patients with low expression of Ets-1 in the tumors. These results also support a role for Ets-1 in tumor progression that is at least in part mediated by increased angiogenesis.

Ets-1 expression has also been shown to correlate with higher microvessel density and a worse prognosis in patients with gastric cancer [31]. Of the 102 patients with primary gastric carcinoma, not involving invasion into the muscularis propria or subserosa, Ets-1 expression was observed in 76% of patients. The 5-year survival rate for these patients was 67% for patients with Ets-1 positive tumors and 89% for those with Ets-1 negative tumors. Ets-1 expression has also been evaluated in patients with colorectal carcinoma [32]. High Ets-1 expression was observed in 48.4% of patients and correlated strongly with increased microvessel density and the expression of VEGF. Similar to the studies in uterine cancer, this study also supports a role for expression of Ets-1 in cancer progression that is at least in part mediated by increased angiogenesis. 

The role of Ets-1 in human brain cancer has also been evaluated. Ets-1 expression was evaluated in 81 primary and 20 recurrent astrocytic tumors [33]. 65% of glioblastomas (grade IV astrocytomas) stained for Ets-1, 25% of anaplastic astrocytomas (Grade III) were positive for Ets-1, and none of the low-grade astrocytomas (Grade II) stained positively for Ets-1. Therefore the expression of Ets-1 was significantly associated with the tumor grade. Normal brain tissue did not express significant amounts of Ets-1 protein. Ets-1 also appears to play a role in regulating tumor angiogenesis in neuroblastomas [35]. Ets-1 expression is higher in undifferentiated human neuroblastomas. Ets-1 expression is stimulated by gastrin-releasing peptide (Grp) that stimulates neuroblastoma growth, and the induction of Ets-1 in these cells promotes the expression and secretion of the angiogenic chemokine interleukin-8. 

The expressions of Ets factors have also been shown to be up-regulated in a number of hematological malignancies either through translocations or through overexpression. One example of an Ets factor that is involved in several different types of hematological malignancies is Tel (ETV6). The hematological malignancies include chronic myeloid leukemia, acute myeloid leukemia, acute lymphoblastic leukemia, and non-Hodgkin's lymphoma [36, 37]. The evidence that angiogenesis plays a pathophysiological role in leukemia has been well documented [38]. Angiogenesis in human leukemias is demonstrated by increased microvessel density (MVD) in the bone marrow, and is associated with the increased expression of hypoxia inducible factor 1*α* (HIF-1*α*), the increased expression of multiple angiogenic factors including VEGF, bFGF, and angiopoetin-2, and a reduction in the expression of endogenous angiogenesis inhibitors such as thrombospondin-1 [39]. Because ETV6 has been shown to be important for yolk sac angiogenesis, its upregulation in several hematological malignancies also suggests that it may likely play an important role in regulating angiogenic factors in hematological malignancies. Furthermore, because several of the Ets factors have been shown to function as downstream effectors of angiogenic factors, targeting ETV6 and other Ets factors in these cancers may not only limit direct tumor cell growth but also may limit the local microenvironment through inhibitory effects on angiogenesis. 

With regard to tumor progression, a number of factors have been shown to promote tumor growth and/or progression. Some of these are directly regulated by Ets factors, while others act synergistically with Ets to regulate specific target genes. One of the earliest events in the development of tumors from epithelial cells is the so-called epithelial to mesenchymal transition (EMT). This process has been associated with increased expression of RhoC, loss of E-cadherin, and acquisition of mesenchymal characteristics. It has recently been shown that the expression of RhoC is regulated by Ets-1 [40]. The tumor stroma is believed to be an important contributor to some of the most malignant characteristics of epithelial tumors. It has recently been shown that targeted deletion of the tumor suppressor PTEN in stromal fibroblasts of mouse mammary glands accelerates the initiation, progression, and malignant transformation of mammary epithelial tumors [41]. More importantly, it was found that loss of PTEN in the stromal fibroblasts was associated with an increase in the expression of Ets-2. The deletion of Ets-2 and PTEN was associated with a decrease in tumor vasculature and the recruitment of macrophages, resulting in fewer and smaller tumors than deleting PTEN alone. There are other recently described links to certain Ets proteins and the development of tumor angiogenesis. For example, the Wilm's tumor suppressor WT-1 was detected in the endothelium of angiogenic blood vessels of 95% of 113 tumors of different origins. Ets-1 and WT-1 have overlapping expression patterns. It was shown that silencing of WT-1 in endothelial cells reduced cell proliferation, cell migration, and endothelial tube formation and that silencing of WT-1 diminished the expression of Ets-1 in the tumors [42]. Mutations of the tumor suppressor gene von Hippel-Lindau (VHL) are associated with highly vascularized tumors that in part have been linked to stabilization of the transcription factor hypoxia-inducible factor (HIF), which upregulates proangiogenic factors such as vascular endothelial growth factor (VEGF). Down regulation of VHL in endothelial cells has recently been linked to Ets-1 activation via increased FGF signal transduction [43]. In Kaposi's sarcoma angiogenesis is associated with the induction of angiopoeitin-2 the expression of which is regulated by the transcription factors AP-1 and Ets-1 [44]. Another important gene for the regulation of tumor angiogenesis, wound healing, and fibrosis is connective tissue growth factor (CCN2). Under pathological conditions, such as wound healing or tumor formation it has recently been shown that Ets-1 competitively inhibits the function of another Ets factor Fli-1 to promote the expression of CCN2, thereby promoting extracellular matrix deposition during cancer [45].

From a therapeutic standpoint, recent studies support important role for Ets factors as critical transcriptional regulators of tumor angiogenesis. Local administration of a dominant-negative form of Ets-1, encoding the DNA binding domain, significantly blocked tumor angiogenesis and tumor growth [46]. Similarly, in a pancreatic mouse cancer model of angiogenesis, the local administration of a dominant negative Ets-1 peptide using and adenoviral vector significantly blunted tumor growth and blood vessel density [47]. By targeting the DNA binding domain, these therapeutic agents very likely target subfamilies of Ets factors, for example, Ets-1 and Ets-2, rather than being entirely selective for a particular Ets factor [7]. 

Administration of dominant negative Ets proteins has not been successfully used to block tumor growth systemically. Another strategy that has been used to inhibit the function of transcription factors is the use of membrane permeable peptides. For example, a peptide with a membrane permeable sequence and the nuclear localization signal sequence of the transcription factor NF-*κ*B can block nuclear import of this transcription factor upon activation [48]. The inhibitory function of this peptide, however, was not specific to NF-*κ*B, and also inhibited other transcription factors such as AP-1, NFAT, and STAT1 from entering the nucleus [49]. Another membrane permeable peptide with the ability to interfere with DNA binding of a transcription factor was recently demonstrated using the Tet repressor (TetR) of *Escherichia coli* synthesized in tandem with a cell membrane transducing peptide. This peptide was able to repress the expression of a tetracycline responsive reporter unit stably transfected into the genome of HeLa cells [50]. Membrane permeable peptides consisting of the terminal portion of the DNA binding domain of the Ets factor Elf-1 in tandem with the HIV TAT peptide readily cross the cell membrane and enter the nucleus of cells where they are able to block the function of the Ets factor Elf-1. These peptides were successfully used to systemically block the growth of B16 melanoma tumor cells in vivo [7]. Furthermore, the identification of relatively short peptides of 30 to 40 amino acids long that can selectively block the DNA binding of specific transcription factors, or subsets of transcription factors, suggests that this may be a very potent novel approach toward targeting transcription factors as a therapeutic modality to inhibit tumor growth and angiogenesis. Although these peptides have been used successfully to block tumor growth in small animals, there may be certain limitations. First the cost of making these peptides may be prohibitive for use on a much larger scale in man. Second, if the peptides are to be used repeatedly, the peptides may be immunogenic and antibodies directed against the peptides may eventually limit their use. With recent advances in drug discovery, the identification and/or development of small molecule inhibitors of subfamilies of Ets factors may be possible in the future by taking advantage of structural similarities among the subfamilies in the DNA binding domain or other functionally conserved regions of the proteins. Although transcription factors have historically been poor drug targets, the advances in computational chemistry may ultimately lead to the identification of selective inhibitors of Ets and other transcription factors. 

## Figures and Tables

**Figure 1:**
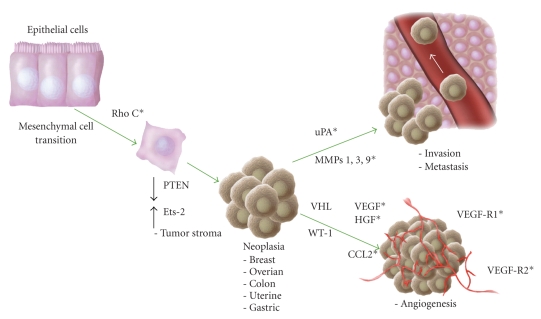
The role of Ets-1 in tumor development, progression, and angiogenesis. The role of Ets-1 is depicted during different stages of neoplasia from the epithelial-mesenchymal transition (EMT) to tumor growth, invasion, metastasis, and tumor angiogenesis. (∗) denotes those genes that are regulated by Ets-1.

**Table 1:** Summary of Ets-1 expression, microvessel density (MVD), and prognosis in human cancer.

Tumor type	Number	% Ets-1 positive*	MVD/VEGF	Prognosis**
(1) Ovarian [24]	30	66	increased MVD	2 years survival:
				10% Ets-1 (+); 60% Ets-1 (−)
(2) Ovarian [25]	30	50	increased MVD	2 years survival:
				30% Ets-1 (+); 70% Ets-1 (−)
(3) Esophageal [26]	55	80	increased MVD	not evaluated
(4) Breast [27]	48	71	increased MVD	not evaluated
(5) Breast [28]	123	62	not evaluated	5 years relapse free survival:
				55% Ets-1 (+); 85% Ets-1 (−)
(6) Endometrial [29]	60	66	increased VEGF	not evaluated
(7) Cervical [30]	60	42	increased MVD	2 years survival:
				54% Ets-1 (+); 92% Ets-1 (−)
(8) Gastric [31]	102	76	increased MVD	5 years survival:
				67% Ets-1 (+); 89% Ets-1 (−)
(9) Colon [32]	95	48	increased MVD	not evaluated
(10) Brain [33]	101	32	not evaluated	not evaluated

“Number” refers to number of patients in the study.

*% refers to percent of primary tumors that express Ets-1.

**% refers to percent of patients that are relapse free at different time points with primary tumors that expressed Ets-1 (+) or did not express Ets-1 (−).

“MVD/VEGF” refers to evaluation of either VEGF expression or microvascular density (MVD) in the tumors
